# Preparation, Properties, and Interaction Mechanism of High-Ratio DCLR-Modified Asphalt

**DOI:** 10.3390/ma18081798

**Published:** 2025-04-15

**Authors:** Lei Xia, Qidong Su, Jian Liu, Qi Wang, Dongwei Cao, Gaoqiang Zhang, Lingyan Shan

**Affiliations:** 1 School of Materials Science and Engineering, Chang’an University, Xi’an 710064, China; 2 Research Institute of Highway Ministry of Transport, Beijing 100088, China; 3 Cangzhou Qugang Expressway Construction Co., Ltd., Cangzhou 062450, China

**Keywords:** road engineering, high-proportion DCLR, composition and structure, multiscale microscopy, high-modulus asphalt, interaction mechanism

## Abstract

In response to the complex pretreatment processes (e.g., solvent dissolution and high-temperature melting) of direct coal liquefaction residue (DCLR) in asphalt, its low-dosage limitation for high-value utilization in asphalt pavement, and the unclear interaction mechanisms between high-proportion DCLR and asphalt, this study comprehensively analyzed the molecular composition and structural characteristics of DCLR at multiple scales using FTIR, GPC, SEM, BET, Tg-FTIR, and XRD. DCLR was crushed to a particle size of 0.15 mm and mixed with 70# base asphalt at mass ratios of 10:100, 15:100, 20:100, 25:100, 30:100, 40:100, and 45:100 at 185 °C to prepare high-proportion DCLR-modified asphalt. The conventional and rheological properties of DCLR-modified asphalt at various dosages were evaluated and compared with those of Buton rock asphalt (BRA)-modified asphalt at equivalent dosages. The results indicated that DCLR and BRA significantly improved the high-temperature performance and PG grade of the base asphalt but reduced its low-temperature performance and grade. At equivalent dosages, DCLR exhibited a more pronounced enhancement in high-temperature performance and a greater reduction in low-temperature performance compared to BRA. High-proportion DCLR-modified asphalt meets the technical requirements for high-modulus asphalt. Using FTIR, GPC, four-component analysis, and elemental analysis, the chemical composition and performance variation trends of high-proportion DCLR-modified asphalt were investigated at multiple scales. The interfacial physical, chemical, and mechanical behaviors between DCLR and base asphalt were characterized. The interaction mechanisms between high-proportion DCLR and asphalt were elucidated, and a novel application strategy for DCLR in asphalt was proposed, significantly enhancing its resource utilization rate in road engineering.

## 1. Introduction

**Figure 1:**
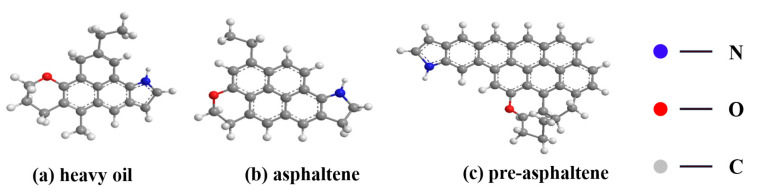
The composition and structure of DCLR.

China’s energy structure is characterized by an abundance of coal but limited oil and gas resources. Coal dominates China’s energy mix and is the primary contributor to carbon emissions. This coal-dominated energy structure has resulted in severe environmental challenges [1]. Direct coal liquefaction, an efficient clean energy conversion technology, holds significant strategic importance for coal resource utilization [2]. However, all coal direct liquefaction processes yield approximately 30% of the raw coal mass as a byproduct, termed direct coal liquefaction residue (DCLR) [3,4]. DCLR is a coal-derived, asphalt-like material characterized by a high hydrogen-to-carbon (H/C) ratio, elevated ash, sulfur, and carbon content, and low volatility. The major components of DCLR include unreacted coal organic matter, inorganic minerals, residual catalyst, and entrained, liquefied heavy oil [5]. DCLR exhibits complex physicochemical properties, consisting of 30% heavy oil, 30% asphaltene, 10% pre-asphaltene, and ~45% tetrahydrofuran insoluble substances [6]. Gu et al. used FTIR, GPC, elemental analysis, and NMR to characterize the structures of heavy oil, asphaltene, and pre-asphaltene (Figure 1), deriving their molecular formulas and weights. The average molecular formula of DCLR was determined as C_25_H_31_O_0.2_N_0.26_, with a molecular weight of 339 [7]. Currently, DCLR utilization primarily relies on gasification, combustion, cracking, and stockpiling. However, these methods are inefficient and underutilized, posing substantial social and environmental hazards [8].

Research has demonstrated that DCLR comprises a significant proportion of polycyclic aromatic hydrocarbons, asphaltene, and other asphalt-like substances. These asphalt-like substances exhibit chemical and performance similarities to Trinidad Lake Asphalt (TLA), characterized primarily by their high aromaticity, elevated carbon content, propensity for polymerization or cross-linking, and potential applicability as asphalt modifiers [9].

Wang et al. demonstrated that modified asphalt with 7% DCLR achieves a penetration grade of 40–55, as specified by the ASTM D5710-95 standard [10]. Zhu reported that a DCLR content of 5–7% enables modified asphalt to meet TMA-50 penetration grade requirements, while a content increase to 10% fulfills TMA-70 requirements [11]. Zhang et al. determined that incorporating 5% molten DCLR at 160 °C optimizes asphalt modification, meeting the technical standard for 50# petroleum asphalt [12]. Zheng modified base asphalt with 7% high-sulfur DCLR, observing a 4 °C increase in the softening point and a 2.93 mm rise in penetration [13]. They observed that the combined incorporation of DCLR and purified DCLR resulted in modified asphalt performance comparable to 5% SBS-modified asphalt. Moreover, the viscosity of the modified asphalt increased with higher DCLR content. Fan proposed that using a matrix asphalt rich in soft components, along with a high shear rate, improves DCLR’s modification effect on petroleum asphalt [14]. Zhao et al. assessed the modification effects of DCLR and rock asphalt at four dosages, discovering that DCLR exerts a stronger influence on asphalt performance compared to rock asphalt, recommending a maximum dosage of 10% [15]. Song et al. independently prepared modified asphalt by extracting individual DCLR components, identifying 1% heavy oil and 4% asphaltene as the optimal composition for effective modification [16]. Chen employed DCLR’s tetrahydrofuran-soluble substance (THFS) to prepare modified asphalt, demonstrating that 4% THFS dosage at 170 °C yielded the optimal modification outcome [17]. Progressing from these findings, Ji et al. proposed that DCLR’s modification of asphalt is a physical process, with THFS content ≤6% resulting in a >10% reduction in G* value [18,19]. Liu et al. established that DCLR enhances asphalt’s resistance to permanent deformation and fatigue cracking [20]. Ji et al. found that DCLR improves the adhesion and water damage resistance between asphalt and limestone [21]. They further investigated the aging effects on THFS-modified asphalt using macroscopic and microscopic methods, recommending a maximum THFS content of 6% [22]. Sheng et al. observed improved anti-aging properties in asphalt following DCLR incorporation, suggesting that the asphaltene content in the asphalt system should not exceed 12.9% [23]. Ji et al. incorporated three distinct compatibilizers into DCLR-modified asphalt and found that silane coupling agent, benzaldehyde, and xylene improved dispersion and tensile flexibility, with limited enhancement in low-temperature crack resistance [24]. Suo et al. reported significant improvements in dynamic stability for DCLR-modified and DCLR-composite-modified asphalt mixtures, particularly at 55–70 °C and under 0.7–1.0 MPa pressure [25]. Continuing this research, Ji demonstrated that the dynamic modulus of DCLR-modified asphalt exceeded those of SK-90 and SBS-modified asphalt mixtures. They developed a rheological parameter model for DCLR-modified asphalt mixture and experimentally validated its high accuracy in rutting prediction [26,27].

Zhang’s research demonstrates that the observed modifications constitute physical processes, manifested through dissolution, adsorption swelling, as well as enhancement and filling effects of DCLR [12]. Wu et al. established that compatibility variations between DCLR and various petroleum asphalts stem from asphaltene effects, as verified by FTIR, four-component analysis, GPC, and atomic force microscopy [28]. Zhao et al. investigated the modification effects of DCLR-lake asphalt blends, demonstrating that DCLR exerts a significantly greater influence on asphalt performance than lake asphalt. The study confirmed that modifications induced by both DCLR and lake asphalt represent primarily physical processes [15].

However, three key limitations persist in current DCLR-asphalt modification research. First, predominant methodologies employ either tetrahydrofuran-soluble fractions (THFS) or molten DCLR (190 °C) for modification. While these pretreatments improve DCLR dispersion, they suffer from low resource utilization, complex extraction, THFS solvent toxicity, low yield, high energy demands, and cost inefficiencies, with potential secondary pollution from residual solvents. Second, both dry and wet application methods demonstrate ≤10% DCLR dosage thresholds, leading to suboptimal utilization efficiency. Third, current mechanistic understanding remains limited to physical modification evidence from performance comparisons and FTIR peak alignment, with inadequate molecular-level analysis of DCLR–asphalt compatibility changes and interaction mechanisms across dosage ratios [29]. Furthermore, structural evolution patterns at >10% DCLR concentrations remain unclear, hindering fundamental solutions for high-value DCLR applications in asphalt.

This study performed comprehensive multiscale analysis (qualitative, quantitative, macroscopic, and microscopic) of DCLR’s molecular composition and structural characteristics employing FTIR, XRD, SEM, BET, and related analytical techniques. High-proportion DCLR-modified asphalts were prepared by blending Aisuo70# asphalt with DCLR at mass ratios ranging from 100:10 to 100:45. The conventional and rheological properties of DCLR-modified asphalts were evaluated and comprehensively compared with Buton rock asphalt (BRA)-modified asphalt at equivalent proportions. Multiscale analysis of chemical composition, structure, and performance was performed for high-proportion DCLR-modified asphalt using FTIR, GPC, four-component analysis, and elemental analysis. The study further investigated the interfacial physical, chemical, and mechanical behavior between DCLR and base asphalt, enabling the development of a microstructure model for high-proportion DCLR-modified asphalt. Furthermore, the interaction mechanisms were elucidated, and high-proportion DCLR application methodology was proposed, significantly improving DCLR resource utilization in road engineering while establishing a novel application approach.

## 2. Materials and Methods

### 2.1. Materials

#### 2.1.1. Asphalt

The base asphalt used in this research is Aisuo70# asphalt produced by Guopei Petrochemical Co., Ltd. in Ningbo, China. The base asphalt was tested according to the “Test Specification for Asphalt and Asphalt Mixtures in Highway Engineering” (JTG E20-2011) [30], detailed in Table 1.

#### 2.1.2. DCLR and BRA

**Figure 2:**
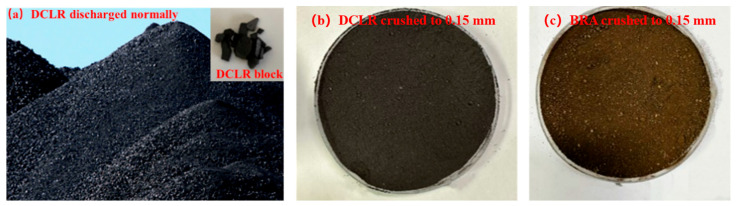
Appearance and morphology of DCLR and BRA.

DCLR, obtained from China Shenhua Coal to Liquid Chemical Co., Ltd., Beijing, China, was ground to a powder of 0.15 mm particle size, depicted in Figure 2a,b. BRA sourced from Buton Rock Asphalt Co., Ltd. in Jakarta Barat, Indonesia is shown in Figure 2c. The basic performance metrics of DCLR and BRA are displayed in Table 2.

The compounds in asphalt are divided by polarity into saturates, aromatics, colloids, and asphaltenes. The rod-shaped thin-layer chromatograph with a hydrogen flame ionization was used to analyze the four-component information of 70# base asphalt, DCLR, and BRA. The results are shown in Table 3.

As indicated in Table 3, the saturation and aromatic contents in DCLR and BRA are significantly lower than in the base asphalt, while the asphaltene contents are exceptionally high at 92.3% and 87.5%, respectively—approximately nine times that of the base asphalt. The differences in colloid content are minimal. Both the saturated and aromatic elements in asphalt belong to the oil component, primarily serving softening and lubricating roles. Conversely, asphaltenes significantly enhance the high-temperature performance of asphalt. Consequently, DCLR and BRA exhibit poor fluidity, impressive high-temperature performance, high viscosity, and tend to be hard and brittle at low temperatures. Specifically, DCLR displays these characteristics more markedly. Colloid stability is gauged by the colloid instability coefficient (Ic), with higher values indicating greater instability. As shown in Table 3, the Ic of the base asphalt was less than 1 with a stable colloid structure, whereas for DCLR and BRA, it reaches 35 and 22.5 times higher than that of the base asphalt, respectively. This indicates that DCLR’s colloid structure is highly unstable, leading to poor integration with the base asphalt.

We used a muffle furnace from Shanghai Yifeng Electric Furnace Co. Ltd. (Shanghai, China) to analyze the moisture, ash, volatile matter, and fixed carbon in the base asphalt, DCLR, and BRA as per the “Industrial Analysis Method of Coal” (GB/T 212-2008) [31]. This standard specifies the methods for determining the moisture, ash content, and volatile matter of coal and coal water slurry, as well as the calculation method for fixed carbon. The results from the industrial and elemental analysis are presented in Table 4.

Vad content serves as a critical indicator of ignition characteristics, with higher content implying easier ignition [32]. The higher the FCad content, the greater the heat generation. According to Table 4, DCLR contains high Vad and FCad levels along with a moderate Aad content. In contrast, base asphalt and BRA demonstrate higher Vad but lower FCad contents. This suggests DCLR contains more inorganic substances, whereas BRA, having the highest Aad content, contains more impurities. Consequently, DCLR is more suitable for modifying base asphalt than BRA. The carbon (C) and hydrogen (H) contents of DCLR and base asphalt were relatively high, with C and H being the predominant components. However, there was a significant difference in the carbon-to-hydrogen ratio (C/H). The C/H ratio of DCLR was approximately twice that of the base asphalt. A higher C/H ratio suggests increased aromaticity in the asphalt, characterized by more cyclic structures within its molecular composition [33]. This suggests that DCLR contains a larger proportion of aromatic cyclic olefins than base asphalt, along with a more complex molecular structure. BRA exhibited significantly lower carbon content and total elemental composition than both DCLR and base asphalt, suggesting a higher impurity content in BRA, which aligns with its elevated ash content.

The high content of S, N, and O elements in DCLR indicated a high content of heteroatoms and strong polarity, while the C/H of BRA was equivalent to that of the base asphalt, with a high content of heteroatoms and more impurities. This indicates that BRA has relatively more straight-chain, branched-chain, and aliphatic alkanes compared to DCLR [34]. Theoretically, while DCLR is more compatible with base asphalt, it adversely affects the asphalt’s low-temperature performance. In contrast, in BRA, aside from impurities like ash that degrade low-temperature performance, its other components are more harmoniously integrated.

### 2.2. The Preparation of Modified Asphalt

**Figure 3:**
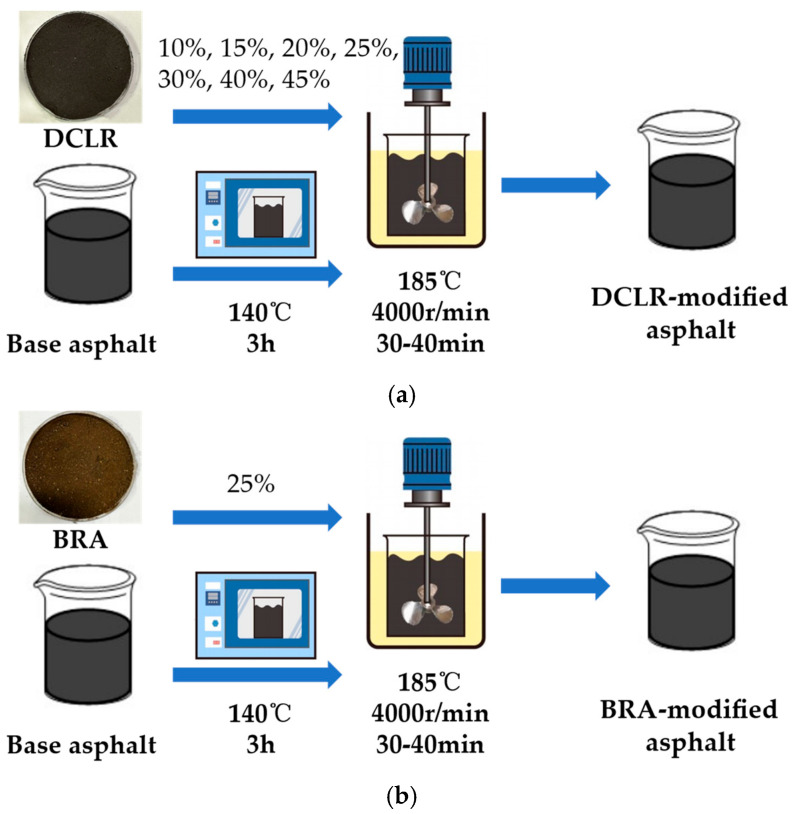
The schematic diagram of different modified asphalt preparation methods. (**a**) The preparation process of DCLR-modified asphalt; (**b**) the preparation process of BRA-modified asphalt.

Place Aisuo 70# base asphalt in an oven preheated to 140 °C and heat for 3 h until it melts. Mix DCLR with the melted base asphalt in mass ratios of 100:10, 100:15, 100:20, 100:25, 100:30, 100:40, and 100:45. Stir the mixture at 185 °C and 4000 r/min for 30–40 min to prepare modified asphalt with varying DCLR content. A schematic diagram illustrating the preparation method for the various modified asphalt is shown in Figure 3.

### 2.3. Asphalt Performance Test

The classical and rheological performance tests were conducted, including penetration, softening point, ductility, the Thin-Film Oven Test (TFOT), the Pressure Aging Vessel accelerated aging test (PAV), and high- and low-temperature grading tests, as well as fatigue level testing in accordance with JTG E20-2011, Standard Test Methods of Bitumen and Bituminous Mixtures for Highway Engineering [30]. The instruments used for asphalt classical performance testing are all from Shanghai Changji Geological Instrument Co., Ltd. (Shanghai, China), located in Shanghai, China. The instruments used for the dynamic shear rheological test (DSR) are from Nuolai Technology Development Co., Ltd., Shanghai, China. The detailed information on the testing conditions and equipment are shown in Table 5.

### 2.4. Composition and Structural Performance Testing

#### 2.4.1. Determination of Four Components of Asphalt

The sample underwent four-component analysis using a rod-shaped thin-layer chromatograph with a hydrogen flame ionization detector (TLC-FID) with model RY-CF19 manufactured by Shandong Runyang Environmental Protection Equipment Co., Ltd., Heze, China. This analysis followed the methods and steps of T0618-1993, as specified in JTG E20-2011, Standard Test Methods of Bitumen and Bituminous Mixtures for Highway Engineering employing solvent precipitation and chromatographic column techniques.

#### 2.4.2. Organic Element Analysis Testing

The elemental analyzer produced by Elementar Analysts GmbH (Frankfurt, Germany) is used for testing elemental composition. The model of the elemental analyzer is Vario EL. The sample underwent catalytic oxidation and decomposition into gas at 1150 °C and 1221 mbar. Helium purchased from Macklin Biochemical Technology Co., Ltd., Shanghai, China was utilized as both the carrier and purge gas, with different gases being separated via adsorption columns. The gases were individually detected using a heat island detector.

#### 2.4.3. Fourier Transformation Infrared Spectroscopy (FTIR) Test

Fourier transform infrared spectroscopy (FTIR) was performed using a Nicolet 6700 produced by Nigel Corporation of the Waltham, MA, USA. The asphalt sample was prepared via the potassium bromide (KBr) pellet method, and the sample was applied evenly to KBr salt plates. DCLR, BRA, and KBr were mixed and ground in a ratio of 1:100, and then pressed into pellets. All samples were scanned 64 times at a resolution of 4 cm^−1^ over a wavenumber range of 400–4000 cm^−1^. The KBr was purchased from Macklin Biochemical Technology Co., Ltd., Shanghai, China.

#### 2.4.4. Gel Permeation Chromatography (GPC) Test

The relative molecular weight distribution of various asphalt samples was determined using gel permeation chromatography (GPC) with an LC20AD system from Shimadzu Corporation, Japan. The mobile phase consisted of tetrahydrofuran (THF), HPLC-grade, supplied by TEDIA Company, Fairborn, OH, USA, with a flow rate of 1.0 mL/min and a column temperature maintained at 35 °C. The standard used was narrow distribution polystyrene (PS) obtained from TOSOH Company, Tokyo, Japan (TSK Dongcao). Approximately 10 mg of the sample was dissolved in 1 mL of THF, thoroughly mixed, and then filtered through a 0.22 µm molecular membrane. A 100 µL aliquot of the filtrate was injected into the GPC system to test the number-average molecular weight (Mn), weight-average molecular weight (Mw), z-average molecular weight (Mz), and MZ+1. The THF was purchased from Macklin Biochemical Technology Co., Ltd., Shanghai, China.

### 2.5. Microscopic Performance Testing

#### 2.5.1. Thermogravimetric Infrared Spectroscopy Combined Method (Tg-FTIR)

The thermogravimetric infrared spectroscopy combined system comprised a Thermo Fisher Mettler thermogravimetric analyzer (TGA) and a Thermo Fisher infrared spectrometer (Is50), connected via a dedicated TGA-FTIR interface (Waltham, MA, USA). The Tg-FTIR is from NETZSCH Instrument Company, Selb, Germany. The interface and transmission line were set at 180 °C. Thermogravimetric analysis was conducted under an argon atmosphere at a flow rate of 50 mL/min, with a circulating water temperature of 20 °C. The temperature ranged from room temperature to 1000 °C at a rate of 10 °C/min, with a sample mass of 30.1540 mg. Fourier transform infrared spectroscopy (FTIR) parameters included a resolution of 4 cm^−1^ and a scanning wavenumber range of 400–4000 cm^−1^.

#### 2.5.2. X-Ray Diffraction Test (XRD)

X-ray diffraction (XRD) analysis was performed using a D8 ADVANCE instrument from Bruker Corporation, Karlsruhe, Germany. A 50 mg sample of dry, finely ground material was spread over the test area, pressed, and placed in the instrument. The tube current and voltage were set to 40 mA and 40 kV, respectively, with a scanning rate of 8°/min and a diffraction angle (2θ) range of 5–80°.

#### 2.5.3. Scanning Electron Microscopy (SEM) Test

For the analysis of the state and microstructure of DCLR and BRA, scanning electron microscopy (SEM) observations were made using an SU8020 microscope from Hitachi Ltd., Tokyo, Japan, operating at an accelerating voltage of 5 kV. Images were captured at a resolution of 50 nm and 5 nm. Before conducting SEM, the surface of the asphalt sample was sputtered using a sputtering instrument to cover it with a layer of gold atomic film with a thickness of about 5–10 nm.

#### 2.5.4. Brunauer–Emmett–Teller (BET) Testing Method

The adsorption isotherm of the sample was measured at 77 K using the ASAP 2460 fully automatic specific surface area and pore size distribution analyzer from Micromeritics Instrument Corp., Norcross, GA, USA. The Brunauer–Emmett–Teller (BET) model calculated the specific surface area, and the Density Functional Theory (DFT) model determined the pore structure.

### 2.6. Research Flowchart

**Figure 4:**
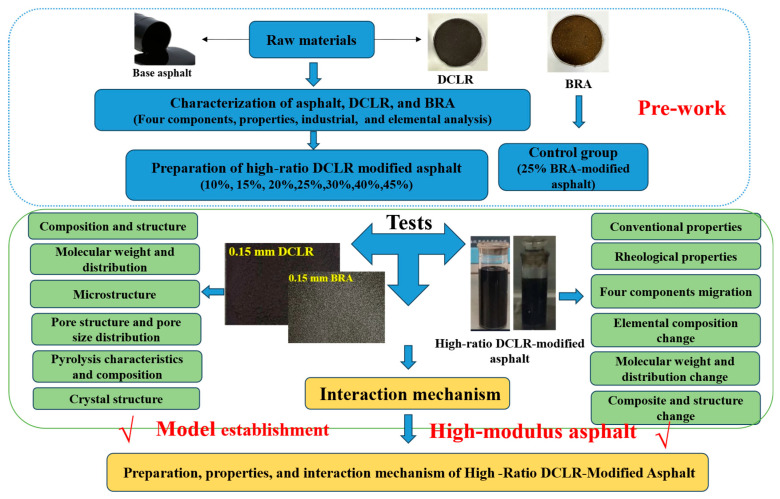
The flowchart of the research.

The testing methods and research process of this study are detailed in Figure 4.

## 3. Results and Discussion

### 3.1. The Composition and Structure of DCLR

#### 3.1.1. FTIR Spectrum Comparative Analysis of DCLR

**Figure 5:**
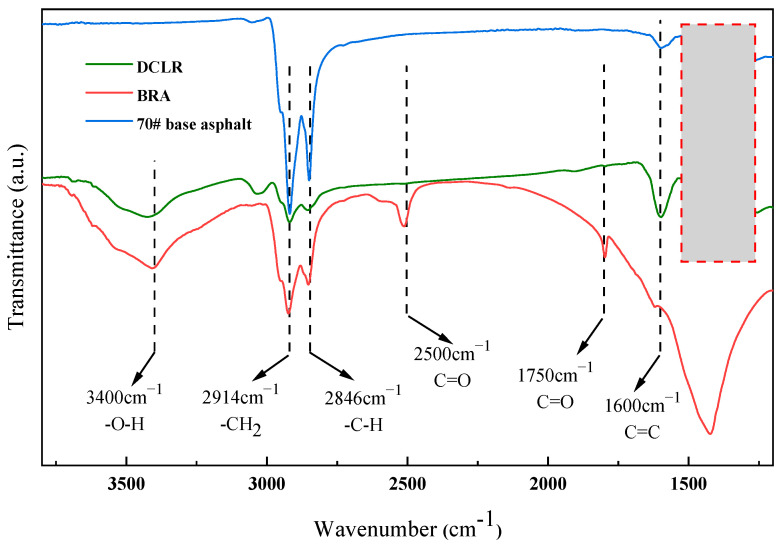
FTIR spectra of DCLR, 70# base asphalt, and BRA.

FTIR analyses were conducted on DCLR, 70# base asphalt, and BRA, and the resulting FTIR spectra are shown in Figure 5.

As shown in Figure 5, the 70# base asphalt and BRA exhibit significant asymmetric stretching vibration peaks corresponding to -CH_2_ and methylene (-C-H) at approximately 2914 cm^−1^ and 2846 cm^−1^, respectively, with the absorption peak of the 70# base asphalt appearing more pronounced. The absorption peak of DCLR at the corresponding peak positions is complex and weak, indicating the presence of numerous straight-chain, branched-chain, and aliphatic hydrocarbons in the 70# base asphalt, which correlates with the lowest saturate of DCLR in the comparative four-component analysis. The C=C stretching vibration peaks associated with the benzene ring skeletons in the double-bond stretching vibration region of 1650–1430 cm^−1^ for DCLR, 70# base asphalt, and BRA are noticeably intense, indicating the presence of single-, multi-ring, and substituted aromatic hydrocarbons. However, the DCLR’s absorption peak is most pronounced around 1650 cm^−1^, indicating the highest proportion of aromatic cyclic olefins; BRA exhibits marked characteristic absorption peaks at 1750 cm^−1^ and 2500 cm^−1^, attributed to non-conjugated -C=O stretching vibrations and peaks characteristic of other oxygen-containing functional groups, suggesting an augmented presence of oxygen-containing impurities, including carboxylic acids, esters, aldehydes, and ketones, in BRA [35]. DCLR and BRA display significant characteristic absorption peaks at approximately 3400 cm^−1^, which are the characteristic absorption peaks of hydroxyl groups in dimers or polymers. The characteristic absorption peaks of DCLR in the 1000–1300 cm^−1^ range within the fingerprint region are exceedingly complex, suggesting that DCLR comprises unsaturated hydrocarbon compounds, including alkane-substituted benzene isomers with unsaturated hydroxyl groups. In contrast, 70# base asphalt possesses fewer characteristic peaks in this region, exhibiting a narrow distribution and predominantly characterized by unsaturated hydrocarbons. The FTIR analysis results are in agreement with the highest levels of oxygen content in BRA and the highest C/H ratio in DCLR, as determined by elemental analysis.

#### 3.1.2. Relative Molecular Weight Distribution of DCLR

**Figure 6:**
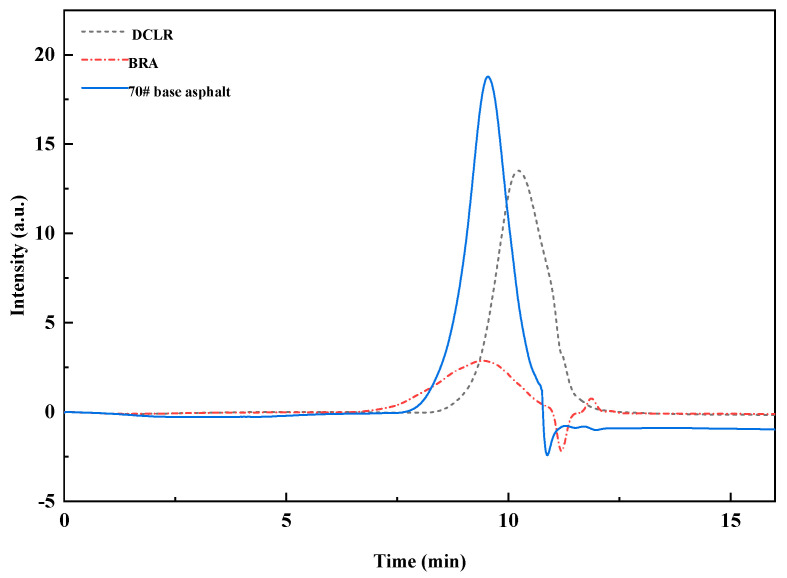
The GPC chromatograms of DCLR, 70# base asphalt, and BRA.

GPC tests were performed on DCLR, 70# base asphalt, and BRA. The GPC chromatograms of different samples are depicted in Figure 6, and the relative molecular weight distributions are presented in Table 6.

Ji et al.’s research shows that significant differences in molecular weight and dispersibility exist between DCLR and BRA [34]. From Figure 6, it is evident that significant differences in molecular weight and dispersibility exist between DCLR and BRA, compared to 70# base asphalt. The 70# base asphalt exhibits the shortest outflow time, followed by BRA, while the absorption peak of DCLR is the last to elute, indicating that 70# base asphalt and BRA contain more macromolecular substances compared to DCLR. Additionally, BRA displays two distinct outflow peaks, suggesting a high dispersibility of BRA substances. According to Table 6, the molecular weight of DCLR is lower than that of 70# base asphalt, featuring an Mn of 471 and a PDI of 1.89, similar to existing research [34]. This suggests that DCLR predominantly consists of low-molecular-weight entities with single dispersibility, while BRA possesses a higher molecular weight compared to 70# base asphalt, indicating a greater presence of high-molecular-weight species. Furthermore, with a PDI of 4.04, BRA exhibits a broader molecular weight distribution and a more complex composition. Consequently, both DCLR and BRA necessitate the application of physical or chemical methods to improve their compatibility with base asphalt, thereby ensuring the homogeneity and stability of the resultant modified system.

### 3.2. Research on Multiscale Microscopic Characteristics of DCLR

#### 3.2.1. Comparative Analysis of Microscopic Morphology of DCLR

**Figure 7:**
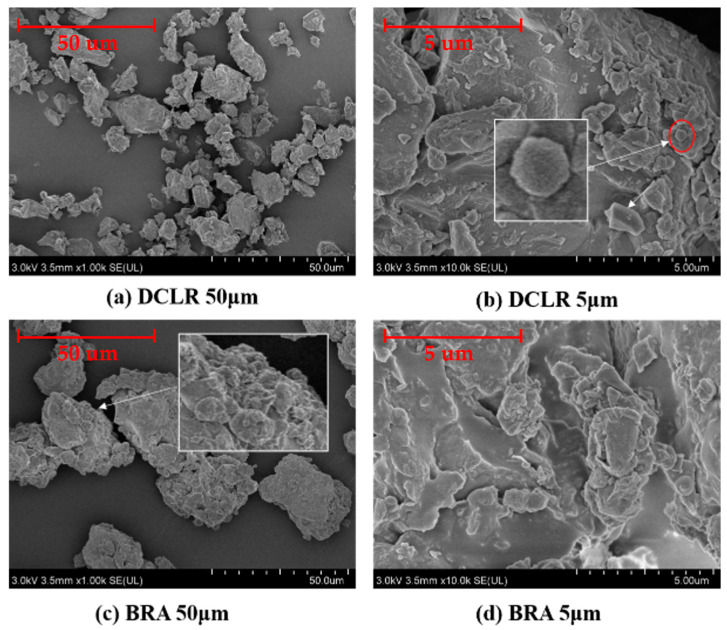
The surface morphology of DCLR and BRA.

The surface morphology of DCLR was examined using SEM, which also investigated DCLR and BRA structures, as illustrated in Figure 7.

Figure 7 reveals a notably compact DCLR structure, exhibiting a variety of morphologies. The macroscopic manifestation is high softening point and complex composition. As shown in the partially enlarged DCLR image in Figure 7b, most of the components of DCLR exist in particle and network structures, mainly manifested as the intermolecular association of asphaltene, heavy oil, and other components in DCLR through functional groups such as hydrogen bonds to form a network structure [36]. The local magnified SEM image of DCLR shows intermediate carbon microspheres with many cross-linked pore structure features, which are intermediate small spheres, indicating the presence of unreacted coal inert components. The internal polycyclic aromatic hydrocarbon structure of DCLR is arranged in a certain orientation in a regular manner, forming a locally larger planar structure. The overall structure of BRA is dense, with a majority of small particle structures, and some parts also have cross-linking and association structures, indicating that there are relatively more aromatic ring structures and inorganic impurities in BRA.

#### 3.2.2. Study on the Pore Structure and Pore Size Distribution of DCLR

**Figure 8:**
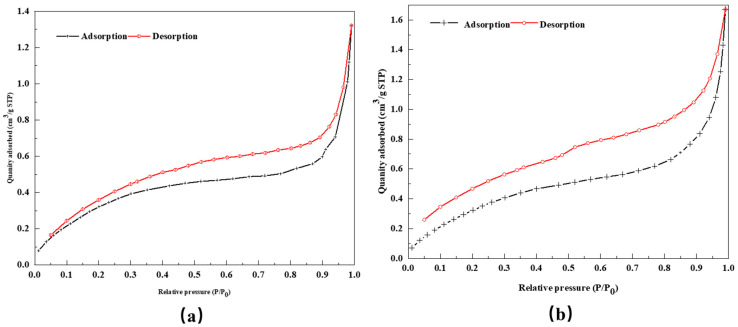
Adsorption/desorption curve: (**a**) DCLR; (**b**) BRA.

BET analysis for specific surface area and pore size distribution was carried out on DCLR and BRA, with results detailed in Table 7. Nitrogen adsorption/desorption isotherms are presented in Figure 8.

As indicated in Table 7, DCLR possesses a higher specific surface area, larger pore volume, and a smaller pore diameter than BRA. This is in line with finding a few cross-linked pore structures in DCLR and the observation of dense small particle structures in BRA’s SEM images. The low-pressure adsorption/desorption isotherms of DCLR and BRA are similar in shape, classified as type IV isotherms. The significant feature of this type of isotherm is the presence of hysteresis loops [37]. The hysteresis loops of DCLR and BRA are similar in shape, and both belong to H3 type, with DCLR having relatively small hysteresis loops. For both DCLR and BRA, adsorption capacity gradually rises when P/P0 < 0.9 and escalates significantly when P/P0 > 0.9. Furthermore, there is no obvious saturation adsorption plateau in the adsorption isotherms of DCLR and BRA, and there is no adsorption saturation in the higher relative pressure region. This suggests that the pore structures and morphologies of DCLR and BRA share similarities, with an irregular internal pore structure, mainly composed of flat slits, cracks, and wedge-shaped structures, typically the narrow-slit pores resulting from sheet-like granular material.

#### 3.2.3. Tg-FTIR Analysis of DCLR

**Figure 9:**
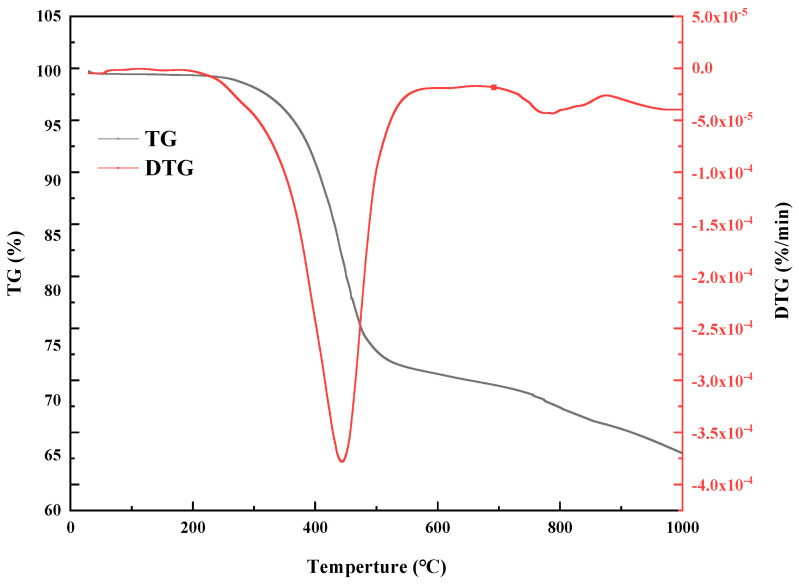
The TG and DTG curves of DCLR.

Figure 9 and Table 8 present the TG and DTG curves and analysis results for DCLR, respectively.

As shown in Figure 9, the pyrolysis of DCLR can be segmented into three stages. The initial stage of DCLR pyrolysis, occurring below 200 °C, primarily involves moisture removal, leading to a minor weight reduction, and the weight loss curve remains basically horizontal. Between 200 and 595 °C, the TG curve exhibits its steepest decline, signifying a drastic mass reduction in DCLR due to substantial volatilization of substances. The DTG curve reveals a pronounced peak within the 200–595 °C range, suggesting that DCLR experiences rapid thermal decomposition, primarily characterized by the volatilization of lighter components like alkanes and aromatics, and the cleavage and polymerization of molecules during pyrolysis, yielding low molecular weight compounds and gases [38]. The reaction peaks in intensity around 448 °C, corresponding to the maximum thermal weight loss rate. The third stage of DCLR pyrolysis commences post 595 °C, characterized by continued weight loss, and begins to stabilize. Shoulder peaks emerge on DCLR’s DTG curve around 740 °C and 900 °C, potentially attributable to mineral decomposition and condensation of DCLR’s organic components.

**Figure 10:**
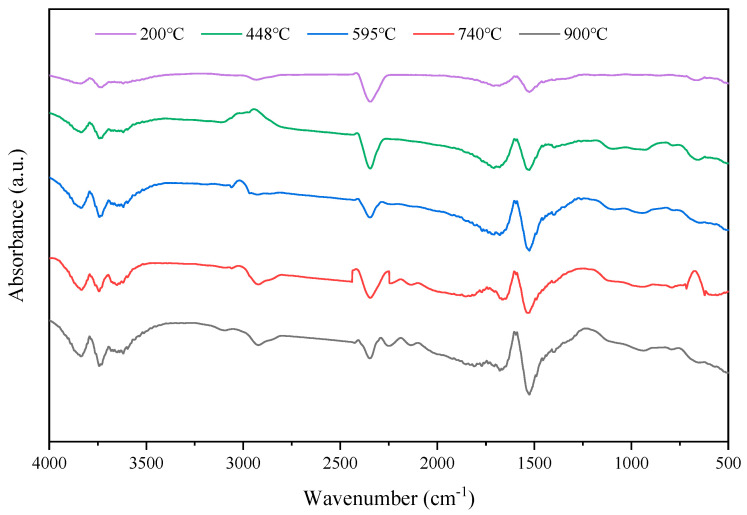
FTIR spectra of volatile substances from DCLR pyrolysis at different temperatures.

Based on the temperature key points and the lag time of pyrolysis gas escape in the DTG curve, the pyrolysis volatiles at 200 °C, 448 °C, 595 °C, 740 °C, and 900 °C were selected for the FTIR test, with results displayed in Figure 10.

In Figure 10, the main release is some small molecule gas compounds, such as CO_2_, SO_2_, and H_2_O at 200 °C. With rising temperatures, an increase in evaporated gas products is noted upon entry into the main stage of pyrolysis. Figure 10 shows small and continuous peaks within the 3500–4000 cm^−1^ range at various temperatures, attributed to the -O-H stretching vibration, signaling the release of H_2_O vapor from small water molecules and the cleavage of -O-H groups on large molecule side chains. Characteristic CO_2_ absorption peaks are observed within the 2240–2400 cm^−1^ range at various temperatures, mainly from the cracking and recombination of carbonyl and carboxyl compounds [39]. Small absorption peaks at 1374 cm^−1^ result from the -S=O bond’s stretching vibration, indicating SO_2_ presence among pyrolysis-generated gas products. Absorption peaks within the 1300–1500 cm^−1^ and 2675–3115 cm^−1^ ranges correspond to -CH_3_, -CH_2_, and -C-H in saturated fatty hydrocarbons. Absorption peaks near 1465 cm^−1^ represent alkyl substitution vibrations in the benzene ring structure, and the absorption vibration peaks at 2926 cm^−1^ belong to -CH_2_, indicating the generation of long-chain alkanes. The absorption peak around 3015 cm^−1^ indicates the release of CH_4_. Figure 9 shows no absorption peak below 448 °C, suggesting that CH_4_ release predominantly results from the cleavage of -O-CH_3_ and -CH_2_ groups. Near 1460 cm^−1^, an absorption peak corresponds to -C-CH_3_ in aromatic cyclic aliphatic compounds, whereas a peak at 1600 cm^−1^ denotes -C=C skeleton vibrations in aromatic rings and their derivatives. The absorption peak around 1700 cm^−1^ is caused by -C-O stretching vibration, while the characteristic absorption peak around 2060–2240 cm^−1^ is mainly generated by CO formed by the fracture of C-O-C and C=O.

#### 3.2.4. XRD Test Analysis of DCLR

**Figure 11:**
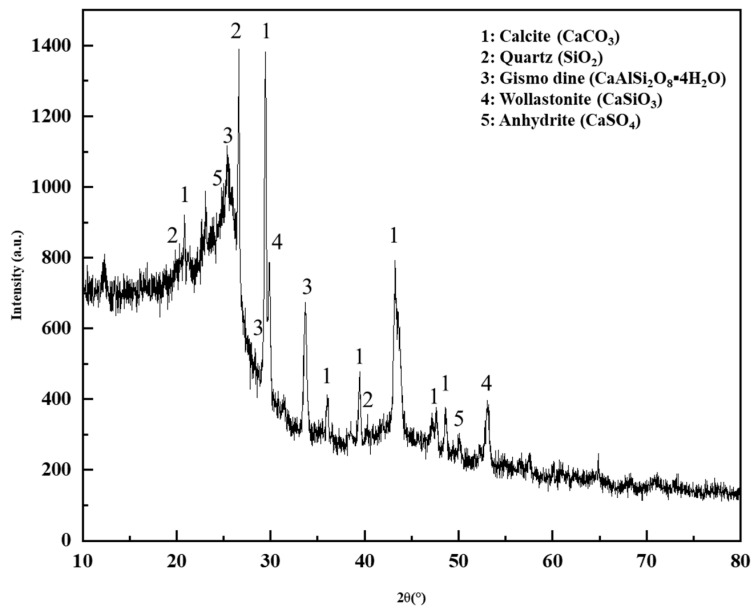
The XRD image of DCLR.

The X-ray diffraction effect occurs in crystals, and the vast majority of minerals are crystals. X-ray diffraction is a crucial and effective method for studying the structure of solid substances. An X-ray diffraction test was performed on DCLR to partially identify the types and forms of minerals in DCLR and unreacted coal, and to analyze the existing forms of catalysts. The XRD image of DCLR is presented in Figure 11.

### 3.3. The Influence of DCLR Content on the Performance of Modified Asphalt

#### 3.3.1. The Influence of DCLR Content on the Conventional Performance of Modified Asphalt

Modified asphalt was prepared by blending DCLR with 70# base asphalt at mass ratios of 100:10, 100:15, 100:20, 100:25, 100:40, and 100:45. Table 9 displays the conventional properties of modified asphalt with different DCLR dosages. The technical requirements for different grades of hard asphalt in the specifications are shown in Table 10.

As indicated by Table 9, incorporating DCLR and BRA markedly enhances the high-temperature performance of base asphalt, evidenced by increased softening points and reduced penetration. DCLR addition notably diminishes the low-temperature performance of asphalt, with a marked decline in ductility observed across DCLR-modified asphalt with different dosages of DCLR. At a 25% dosage level, DCLR presents a more considerable enhancement to the high-temperature performance and a greater reduction in low-temperature performance than BRA. Before and after aging, the 25% BRA-modified asphalt exhibits significantly higher ductility and a lower softening point than the 25% DCLR-modified asphalt. This is primarily because DCLR contains more asphaltenes, fewer aromatic and saturated components, and does not alter the quartet composition of asphalt when blended with base asphalt.

Per Table 9 and Table 10, the 25% BRA-modified asphalt’s performance completely satisfies the 35# hard asphalt criteria specified in “Hard Road Petroleum Asphalt” (GB/T 38075) [40]. With the exception of ductility, the 25% and 30% DCLR-modified asphalt fulfill the 25# hard asphalt standards outlined in “Petroleum Asphalt for Hard Roads” (GB/T 38075). The 40% DCLR-modified asphalt conforms entirely to the 15# hard asphalt specifications in “Hard Road Petroleum Asphalt” (GB/T 38075), while the 45% DCLR-modified asphalt meets the same standards except for the ductility. The corresponding amount of DCLR-modified asphalt meets the technical requirements of the corresponding grade of high-modulus asphalt in the “Technical Guidelines for Construction of High Modulus Asphalt Pavements on Highways” (DB13T2823-2018) [41]. This conclusively shows that the structural and compositional traits of DCLR can be thoroughly harnessed to produce high-modulus asphalt by adding DCLR in a high proportion, which can significantly save material costs.

For DCLR particles (0.15 mm) at content below 15%, the segregation-induced softening point variation in DCLR-modified asphalt remains within 2.5 °C, complying with standard specifications. At 20% DCLR content, this variation increases to 2.7 °C, exhibiting a concentration-dependent increase that reaches 8.5 °C at 45% DCLR content. Comparative analysis reveals that at equivalent 25% concentrations, BRA-modified asphalt shows approximately double the softening point variation of DCLR-modified asphalt, demonstrating poorer dispersion stability in the asphalt matrix.

This phenomenon originates from substantial compositional disparities in the four components among DCLR, BRA, and base asphalt, where conventional mixing processes (stirring, swelling, shear dispersion) cannot establish stable colloidal equilibrium [35]. Furthermore, both modifiers contain inorganic ash components with higher density than base asphalt, with higher concentrations promoting sedimentation due to density differentials. BRA’s greater density accounts for its more pronounced physical filling, distinct phase interfaces, and enhanced segregation tendency at equivalent concentrations.

Therefore, for high-concentration DCLR applications (>15%), dry direct feeding is recommended to prevent segregation and facilitate high-modulus asphalt mixture production.

#### 3.3.2. The Influence of DCLR Content on the Rheological Properties of Modified Asphalt

TFOT and TFOT+PAV tests were conducted on DCLR-modified asphalt, and DSR was used to conduct high-temperature grade tests. A bending beam rheometer was employed for the low-temperature grade test of DCLR-modified asphalt, with the outcomes detailed in Table 11.

Table 11 indicates that the high-temperature grades of 10%, 15%, 20%, and 25% DCLR MA have risen from 58 °C to 70 °C relative to the 70# base asphalt. What’s more, for DCLR contents exceeding 25%, the high-temperature grades of 30% to 45% DCLR MA have been elevated by two grades when compared to the 70# base asphalt. At equivalent dosages, BRA and DCLR exhibit similar enhancements in the high-temperature grades of modified asphalt, both significantly boosting high-temperature performance. For DCLR contents below 25%, the low-temperature grades of DCLR-modified asphalt are downgraded by one grade in comparison to the 70# base asphalt. For DCLR contents above 25%, the low-temperature grades of DCLR-modified asphalt rise from −16 °C to −10 °C. The low-temperature grade of 25% BRAMA surpasses that of 25% DCLRMA by one grade, suggesting a substantial decline in the low-temperature performance of DCLR-modified asphalt as DCLR content rises. In comparison to BRA, DCLR exerts a more pronounced effect on the low-temperature performance of asphalt.

### 3.4. Composition and Microstructure of High-Proportion DCLR-Modified Asphalt

#### 3.4.1. High-Proportion DCLR-Modified Asphalt Component Recombination

**Figure 12:**
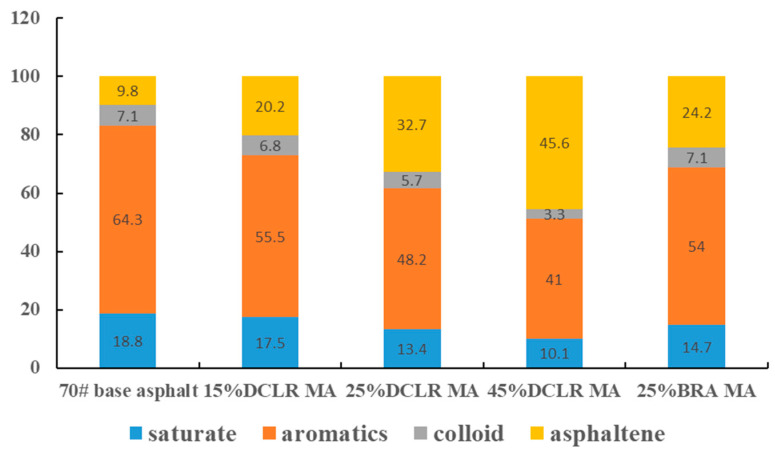
Component migration changes of DCLR-modified asphalt.

Four-component analysis was performed on 70# base asphalt, 15% DCLR MA, 25% DCLR MA, 45% DCLR MA, and 25% BRA MA. The composition of the four components is depicted in Figure 12.

As depicted in Figure 12, an increase in DCLR content results in a gradual decline of saturation, aromatics, and colloid content in the modified asphalt, accompanied by a rise in asphaltene content. This suggests that the incorporation of DCLR reduces the light components within the asphalt system. Some of these light components may be released as gas during the high-temperature preparation of modified asphalt, while others may migrate to the asphaltene of the system, leading to the recombination of asphalt components. Specifically, as DCLR content increases, the softening point of the asphalt progressively rises, and ductility correspondingly diminishes. At equivalent dosages, the addition of BRA causes a minor migration of asphalt saturation and aromatics to asphaltene, with colloid content remaining stable. The reduction rate of saturation and aromatics is less significant than with DCLR, primarily due to BRA’s C/H ratio being similar to that of the base asphalt, whereas DCLR’s C/H ratio is over double that of the base asphalt. This microscopic analysis fully elucidates why, at the same dosage, BRA has a less pronounced effect on the low-temperature performance of asphalt than DCLR.

#### 3.4.2. Research on the Element Composition of High-Ratio DCLR-Modified Asphalt

Elemental analysis was performed on 70# base asphalt, 15% DCLR MA, 25% DCLR MA, 45% DCLR MA, and 25% BRA MA, with the findings detailed in Table 12.

Table 11 indicates that the carbon and hydrogen content in various DCLR and BRA-modified asphalts remains notably high, with C and H constituting the predominant components. The C/H ratio gradually shrinks to a level equivalent to the base asphalt, which indicates that the asphaltene and aromatic components in the modified system are reduced, and the system components undergo reaction and reorganization. As DCLR content increases, the C and H contents diminish, with the decline in C content being more pronounced than that of H. At equivalent dosages, the C/H ratio of 25% BRA MA aligns more closely with that of the base asphalt, and the reduction in C content is less significant. This discrepancy is attributable to the higher impurity content and lower C content inherent in the components of BRA. In addition to impurities impacting the low-temperature performance of asphalt, other components within BRA exhibit greater compatibility with the base asphalt.

#### 3.4.3. The Molecular Weight Distribution of High-Proportion DCLR-Modified Asphalt

**Figure 13:**
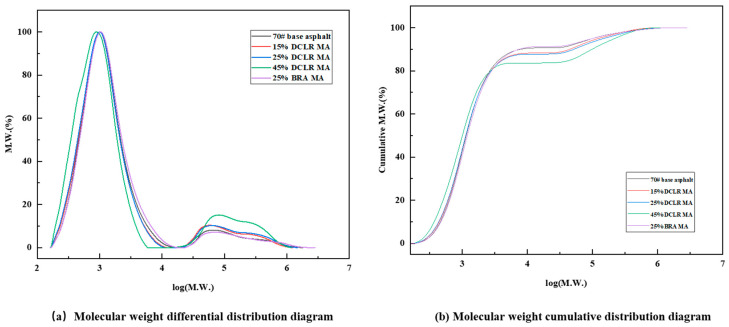
The molecular weight distribution curve of different asphalts.

GPC analysis was performed on 70# base asphalt, 15% DCLR MA, 25% DCLR MA, 45% DCLR MA, and 25% BRA MA. The molecular weight distribution curves for various samples are presented in Figure 13.

As shown in Figure 13a, the molecular weights of 70# base asphalt, DCLR-modified asphalt with different dosages of DCLR, and BRA-modified asphalt are concentrated at around 1000, with the minimum and maximum molecular weights above 160 and 250,000, respectively; a proportional increase phenomenon occurs between 30,000 and 60,000 molecular weights. An increase in DCLR content leads to a rise in the proportion of small molecules with molecular weights less than 1000. Among them, BRA has the least impact on the molecular weight of the system, indicating that the addition of DCLR gradually increases the content of small molecules. Specifically, the number-average molecular weight (Mn) declines as DCLR content rises, whereas the Mn of 25% BRA MA matches that of the base asphalt. With an increase in DCLR content, the proportion of molecules with moderate weights (1000–31,000) diminishes, whereas the moderate molecular content of 25% BRA MA surpasses that of 70# base asphalt. This suggests that some large molecular structures in DCLR, particularly those with active -OH and other oxygen-containing functional groups in condensed aromatic ring structures, have engaged in partial chemical reactions with the active hydrogen in the asphalt, yielding small molecule substances and thereby increasing the concentration of molecules below 1000 molecular weight in the DCLR-modified system.

However, the cyclic structures of moderate molecular weight, characteristic of aromatic components, are relatively stable and undergo component recombination primarily through adsorption and swelling. The molecular weight distribution of BRA closely resembles that of 70# base asphalt, and BRA has a lesser effect on small molecules within the modified asphalt system compared to DCLR. An increase in DCLR content results in a gradual rise in the proportion of molecules, with molecular weights exceeding 31,000, signifying an increase in the system’s large molecular structures. This is mainly due to the component migration of the high proportion of asphaltene in DCLR, while BRA, which has a similar molecular weight distribution to the base asphalt, exhibits a change in molecular weight distribution consistent with the base asphalt.

Figure 13b shows that for molecular weights below 2500, the cumulative distribution of molecular weight rises as DCLR content increases, suggesting that higher DCLR content correlates with more vigorous chemical reactions involving active groups. For molecular weights above 2500, the migration of asphaltene components becomes more pronounced. As DCLR content increases, the cumulative distribution of molecular weight gradually declines, and the cumulative distribution curve for the molecular weight of BRA-modified asphalt closely aligns with that of the base asphalt. This suggests that fewer small molecule reactions occur between BRA and the asphalt system, and the similar molecular weight distribution leads to relatively less migration of components with varying molecular weights.

#### 3.4.4. FTIR Spectrum Analysis of High-Proportion DCLR-Modified Asphalt

**Figure 14:**
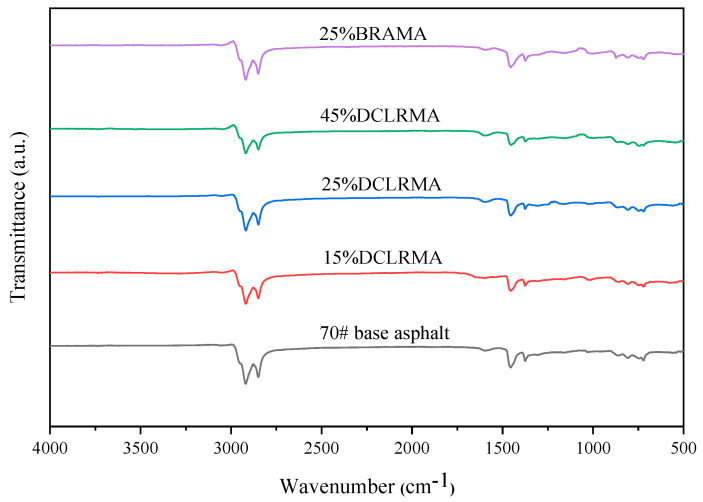
The FTIR spectra of DCLR-modified asphalt with different dosages of DCLR.

We chose 70# base asphalt, 15% DCLR MA, 25% DCLR MA, 45% DCLR MA, and 25% BRA MA with more pronounced penetration characteristics for comparative analysis. FTIR analysis was conducted on 70# base asphalt, 15% DCLR MA, 25% DCLR MA, 45% DCLR MA, and 25% BRA MA, with the resulting FTIR spectra illustrated in Figure 14.

Figure 14 reveals that the infrared spectra of DCLR-modified asphalt and BRA-modified asphalt are largely congruent with that of the base asphalt but compared with the infrared spectra of DCLR and BRA, the characteristic absorption peak intensity of hydroxyl groups in dimers or polymers at around 3400 cm^−1^ significantly decreases or disappears. Relative to BRA, 25% BRA MA exhibits a significant decrease in the characteristic absorption peaks of non-conjugated -C=O stretching vibration peaks and oxygen-containing functional groups at 2500 cm^−1^, indicating that the oxygen-containing or unsaturated functional groups in DCLR and BRA react with active hydrogen and other substances in the base asphalt at around 185 °C to release small-molecule active substances such as H_2_O, CO_2_, SO_2_, and bring about the recombination of the modified asphalt components. This is consistent with the pyrolysis gases of H_2_O, CO_2_, and SO_2_ in DCLR at 200 °C in Tg-FTIR analysis, and further explains the molecular weight distribution changes of different dosages of DCLR-modified asphalt from a molecular structure perspective.

Figure 14 demonstrates that as DCLR content increases, the intensity of the asymmetric stretching vibration peaks of -CH_2_ and methylene -C-H at 2914 cm^−1^ and 2846 cm^−1^ diminishes, while the stretching vibration peak of the benzene ring skeleton -C=C within the double-bond stretching vibration region of 1650–1430 cm^−1^ is comparatively heightened. This is due to the low saturation content, extremely high asphaltene content, fewer aliphatic alkanes, and more aromatic cyclic olefin structures in DCLR when added to the base asphalt in a high proportion, resulting in component recombination. It is evident that the addition of a high proportion of DCLR to the base asphalt partially expedites the recombination of the modified asphalt components. Meanwhile, DCLR, being a granular microporous material with a specific surface area, experiences dissolution, adsorption, swelling, dispersion, and filling effects within the asphalt system. A network structure model of asphalt microcomponents, including an interfacial transition layer, is established under the influence of molecular forces such as van der Waals forces, surface tension, and interfacial stress.

### 3.5. The Interaction Mechanism Between High-Proportion DCLR and Asphalt

**Figure 15:**
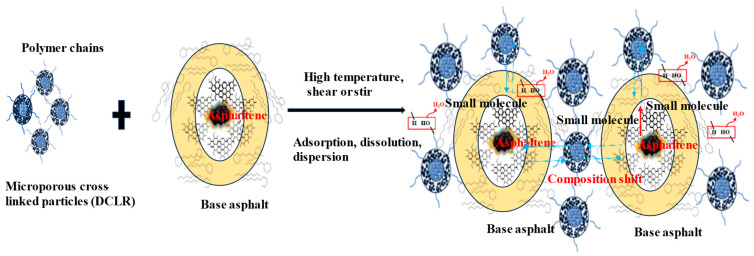
The schematic diagram of the interaction mechanisms between DCLR and base asphalt.

Figure 15 illustrates the schematic diagram of the interaction mechanisms between a high proportion of DCLR and base asphalt.

Research shows swelling treatment could significantly change the surface morphology and pore structure of DCLR, resulting in the increase in pores in the residue [35]. After adding DCLR to the base asphalt, the first step is to absorb the saturated and aromatic components in the base asphalt under high temperature and stirring or shearing action. The DCLR dissolves and softens gradually, which opens up its highly associated network structure, leading to partial polymer chain breakage. DCLR then swells and loosens with some degree of adhesion, becoming uniformly dispersed within the base asphalt.

The active -OH and additional functional groups within DCLR’s partially condensed aromatic ring structures react with active hydrogen in the asphalt, forming small molecules. This furthers DCLR’s swelling and hastens the migration of modified asphalt components.

After DCLR absorbs the base asphalt’s saturated and aromatic components, the original colloidal structure of the base asphalt undergoes change. The content of colloid and asphaltene increases, the similar components that occur in base asphalt and DCLR migrate, permeate, and establish a new colloidal structure system. The ash and some macromolecular substances contained in DCLR cannot be dissolved in the base asphalt; they were formed in a network structure mode under molecular forces such as van der Waals force, surface tension, and interface stress through dispersion, filling, and reinforcement, with a macroscopic manifestation of an increase in hardness and a significant improvement in high-temperature performance.

### 3.6. Application of High-Ratio DCLR-Modified Asphalt

Building upon established dry-process DCLR research, contents exceeding 15% can be incorporated directly into asphalt mixtures via dry processing, replacing the corresponding proportion of base asphalt or modified asphalt for high-modulus asphalt concrete applications.

**Figure 16:**
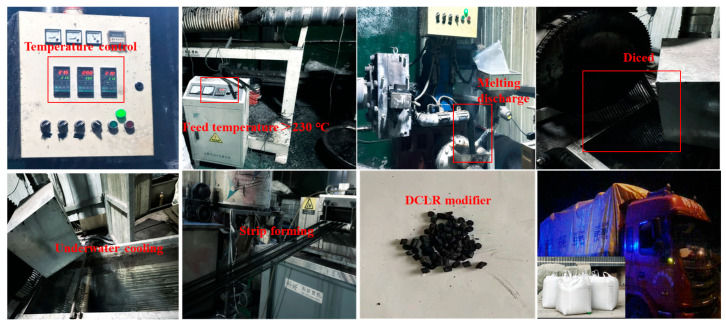
The preparation process of DCLR modifier particles.

DCLR is refined through mixing and then melted and granulated at high temperature using a twin-screw extruder to prepare DCLR modifier particles. Then, DCLR modifier particles are added to prepare the mixture, with a DCLR modifier particle content of 20% of the SBS modified asphalt content. The oil–stone ratio of the mixture is 4.8%. The preparation process of DCLR modifier particles is shown in Figure 16.

According to the “Technical Specifications for Construction of Highway Asphalt Pavements” (JTG_F40-2004) [42], the mix design is carried out to determine the optimal gradation and asphalt aggregate ratio. Standard asphalt mixture specimens were prepared, and key performance tests were conducted. The test results are shown in Table 13.

**Figure 17:**
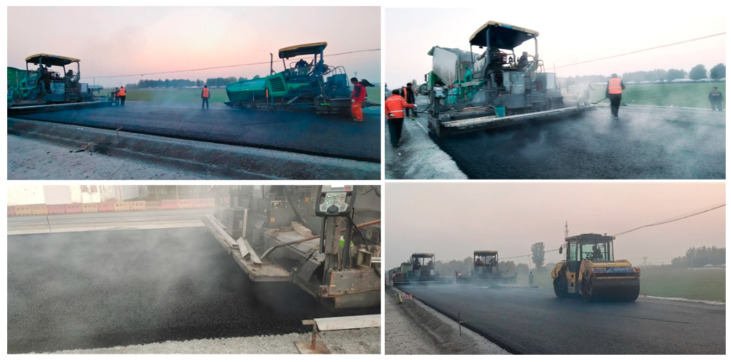
Laying and rolling of high-ratio DCLR-modified asphalt mixture.

**Figure 18:**
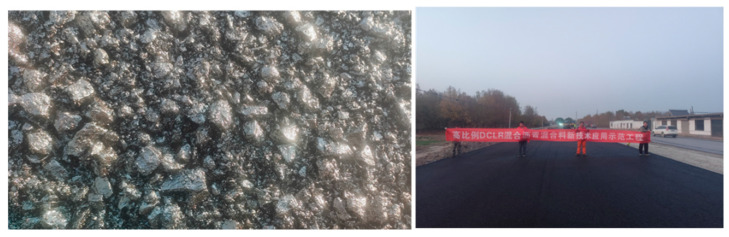
High-proportion DCLR-modified asphalt mixture pavement.

According to Table 1, high-ratio DCLR-modified asphalt mixture has excellent high- and low-temperature performance. Its dynamic stability is 8580 times/mm, effectively solving the problem of the segregation of high-ratio DCLR in asphalt. In addition, a high ratio of DCLR has been successfully utilized to significantly improve the high-temperature performance of asphalt mixtures. Following this technical approach, this research constructed a test road in actual engineering, as shown in Figure 17 and Figure 18.

## 4. Conclusions

The molecular composition and structure characteristics of DCLR were systematically investigated using multiscale microscopic characterization, and multiple DCLR-modified asphalt formulations were prepared by blending with 70# base asphalt at varying proportions. The conventional and rheological properties of high-proportion DCLR-modified asphalt were comprehensively evaluated and compared. Interfacial physical, chemical, and mechanical behavior between DCLR and base asphalt were examined through microscopic analysis. A microstructure model for high-proportion DCLR-modified asphalt was established, and the interaction mechanisms of high-proportion DCLR with asphalt were elucidated. The key findings can be summarized as follows:

(1) DCLR and BRA exhibit significantly lower saturated and aromatic fractions compared to base asphalt, but substantially higher asphaltene content. DCLR comprises unsaturated hydrocarbon compound, including alkane-substituted benzene isomer, featuring unsaturated hydroxyl groups and an elevated C/H ratio. Its ash composition contains abundant heteroatoms (e.g., SiO_2_, CaSO_4_, Al_2_(SO_4_)_3_), contributing to strong polarity and colloidal instability. DCLR possesses a compact structure where asphaltene and heavy oil form a microporous, cross-linked network via hydrogen-bonding functional groups, exhibiting irregular pore morphology.
(2) Both DCLR and BRA additives significantly improve the base asphalt’s high-temperature performance and PG grade, while reducing its low-temperature performance and PG grade. At equivalent dosages, DCLR demonstrates greater enhancement of high-temperature performance and more significant deterioration of low-temperature performance relative to BRA. The 40% high-proportion DCLR-modified asphalt formulation satisfies all technical specifications for high-modulus asphalt applications.
(3) Under elevated temperatures with mechanical shear, DCLR absorbs asphalt’s light components (saturated and aromatics), undergoing dissolution and swelling that disrupts its highly associated network structure. Below 200 °C blending temperature, reactive -OH groups and other functionalities in DCLR’s condensed aromatic rings interact with asphalt’s active hydrogen, producing gaseous byproducts (CO_2_, SO_2_, and H_2_O). This process enhances DCLR swelling, accelerates components’ migration/recombination, and ultimately establishes a new modified colloidal system. At the macroscopic level, this modification results in increased hardness and substantially enhanced high-temperature performance. The newly formed colloidal equilibrium remains stable at DCLR concentrations below 15% (0.15 mm particle size). However, at DCLR concentrations exceeding 15%, increasing insolubility occurs in the matrix asphalt owing to DCLR’s ash content and high-molecular-weight components. Ultimately, density differences destabilize this colloidal system, leading to sedimentation and phase separation.
(4) Building upon established dry-process DCLR research, contents exceeding 15% can be incorporated directly into asphalt mixtures via dry processing, replacing the corresponding proportion of base asphalt or modified asphalt for high-modulus asphalt concrete applications. Subsequent experimental validation of these mixture formulations is recommended.

## Figures and Tables

**Table 1:** Base properties of AiSuo 70# road petroleum asphalt.

Properties	Technical Requirements	Value
Penetration (25 °C) (0.1 mm)	60~80	64.3
Softening point (°C)	≥45	47.8
Ductility (15 °C) (cm)	≥100	>100
Dynamic viscosity (60 °C) (Pa·s)	≥160	185
Mass loss (%)	≤±0.8	−0.403
Penetration ratio (25 °C) (%)	≥61	64.7
Ductility (10 °C) (cm)	≥6	8

**Table 2:** The basic performance of DCLR and BRA.

Properties	DCLR	BRA
Ash content (%)	13.02	35
Penetration (25 °C, 100 g, 5 s) (0.1 mm)	2	2
Softening point (5 °C) (°C)	180	160
Mass loss (%)	0.374	0.58
Flash point (°C)	330	300
Density (25 °C) (g/cm^3^)	1.211	1.76
Water content (%)	1	0.2
Solubility (%)	48.5	45

**Table 3:** The four-component composition of 70# base asphalt, DCLR, and BRA.

Specimen	ω (Saturate) (%)	ω (Aromatics) (%)	ω (Colloid) (%)	ω (Asphaltene) (%)	Colloid Unstable Coefficient (Ic)
DCLR	1.1	3.9	2.7	92.3	14.15
BRA	2.5	7.8	2.2	87.5	9
70# base asphalt	18.8	64.3	7.1	9.8	0.4

Note: Ic = (asphaltene + saturate)/(colloid + aromatics).

**Table 4:** The results of industrial and elemental analysis of 70# base asphalt, DCLR, and BRA.

Specimen	Industrial Analysis, m (%)				Elemental Analysis, m (%)					
	Mad	Aad	Vad	FCad	C	H	S	N	O *	C/H
DCLR	0.08	14.24	34.63	51.05	78.76	4.036	1.768	1.63	9.985	19.51
BRA	0.77	48.91	50.32	0	26.54	2.255	2.29	0.95	27.753	11.77
70# base asphalt	0.43	0.15	82.91	16.51	84.3	9.083	4.874	0.47	1.145	9.28

Note: * subtraction calculation.

**Table 5:** Testing conditions and instrument information for asphalt.

Test Items	Instrument Model	Test Conditions
Softening point	SYD-2806F	5 °C
Ductility	SYD-4508C	5 cm/min, 25 °C or 15 °C
Penetration	SYD-2801E1	25 °C, 100 g, 5 s
TFOT	SYD-0610	15 r/min, 4000 mL/min, 163 °C
PAV	PAV-1	2.1 Mpa, 20 h, 50 g
Brookfield viscosity	NDJ-1C	135 °C
DSR	DHR-10	Strain value: 12%, angular frequency: 10 rad/s

**Table 6:** The relative molecular weight distribution.

Specimen	Mn	Mw	Mz	Mz+1	PDI
DCLR	248	471	1023	2051	1.89
BRA	970	3922	16,059	35,417	4.04
70# base asphalt	874	1481	2766	4860	1.69

**Table 7:** The specific surface area and pore size distribution of DCLR and BRA.

Specimen	BET Surface Area (m^2^/g)	t-Plot Pore Volume (cm^3^/g)	Pore Diameter (Å)
DCLR	14.352	0.000404	37.111
BRA	1.5082	0.000276	51.143

**Table 8:** Thermal analysis results of DCLR.

Specimen	Pyrolysis Stage	Initial Temperature (°C)	Maximum Peak Temperature (°C)	Final Pyrolysis Temperature (°C)	Weight Loss Rate (%)
DCLR	3	200	448	595	34

**Table 9:** The conventional properties of modified asphalt with different DCLR dosages.

Properties		DCLR Content							BRA Content
		10%	15%	20%	25%	30%	40%	45%	25% BRA
Softening point (5 °C) (°C)		52.5	55.5	57	58.5	59.5	61.3	64.5	55.7
Penetration (25 °C, 100 g, 5 s) (0.1 mm)		39	35	31	26	23	19	16	33.5
Ductility (5 cm/min, 25 °C) (cm)		69	40	25	16	14	10	7	54.6
Ash content (%)		1.5	1.7	2.4	3.1	3.8	4.1	4.5	3.9
Brookfield viscosity (135 °C) (Pa·s)		0.56	0.613	0.693	0.752	0.826	0.975	1.256	0.915
Density (15 °C)		1.052	1.066	1.103	1.081	1.130	1.145	1.157	1.081
Segregation (°C)		0.5	1.2	2.7	3.8	5.2	7.1	8.5	7.4
After TFOT	Mass loss (%)	−0.403	−0.264	−0.122	−0.01	−0.087	−0.062	−0.023	0.197
	Penetration ratio (25 °C) (%)	63	65	68	73	77	74	81	81
	Ductility (5 cm/min, 25 °C) (cm)	15	11	10	8	6	5	4	42.5

**Table 10:** Technical requirements for different grades of hard asphalt in the specifications.

Properties		Grade of Asphalt		
		HA-15	HA-25	HA-35
Softening point (5 °C) (℃)		≥60	≥57	≥55
Penetration (25 °C, 100 g, 5 s) (0.1 mm)		10–20	20–30	30–40
Ductility (5 cm/min, 25 °C) (cm)		≥10	≥30	≥50
Ash content (%)		-	-	-
Brookfield viscosity (135 °C) (Pa·s)		-	-	-
Density (15 °C)		-	-	-
After TFOT	Mass loss (%)	≤±0.3	≤±0.3	≤±0.4
	Penetration ratio (25 °C) (%)	≥70	≥67	≥65
	Ductility (5 cm/min, 25 °C) (cm)	-	-	-

**Table 11:** Temperature grade of DCLR-modified asphalt.

Specimen	70# Base Asphalt	10% DCLR MA *	15% DCLR MA	20% DCLR MA	25% DCLR MA	30% DCLR MA	40% DCLR MA	45% DCLR MA	25% BRA MA
PG grading	58–22	70–16	70–16	70–16	70–10	76–10	76–10	76–10	76–10

Note: * modified asphalt (MA).

**Table 12:** The element composition of high-ratio DCLR-modified asphalt.

Specimen	C (%)	H (%)	S (%)	N (%)	O * (%)	C (%)
70# base asphalt	84.3	9.083	4.874	0.47	1.145	9.28
15% DCLR MA	83.26	8.444	4.487	0.52	2.858	9.86
25% DCLR MA	82.84	8.162	4.355	0.52	3.828	10.15
45% DCLR MA	81.22	6.779	3.576	0.65	7.07	11.98
25% BRA MA	76.54	8.237	4.637	0.48	8.524	9.29

Note: * calculation by subtraction.

**Table 13:** Performance test results of asphalt mixtures.

Properties	Technical Requirement	Test Result
Marshall stability (kN)	≥8	15.16
Flow value (mm)	2~4.5	3.58
Residual stability (%)	≥85	95.4
Freeze–thaw splitting strength ratio (%)	≥80	90.7
Dynamic stability (times/mm)	≥3600	8580
Permeability coefficient (mL/min)	≤120	36
Low-temperature bending failure strain (με)	≥2500	2882

## Data Availability

The original contributions presented in this study are included in the article. Further inquiries can be directed to the corresponding author.
